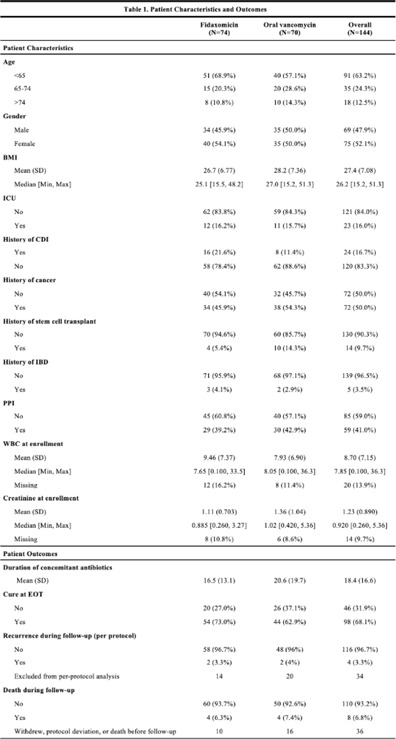# Comparison of fidaxomicin to oral vancomycin for the treatment of Clostridioides difficile infection in hospitalized patients

**DOI:** 10.1017/ash.2022.195

**Published:** 2022-05-16

**Authors:** A. Krishna Rao, Qianzi Zhao, Jay Krishnan, Justin Bell, Oryan Henig, Jolene Daniel, Kara Sawaya, Owen Albin, John Mills, Lindsay Petty, Kevin Gregg, Daniel Kaul, Anurag Malani, Jason Pogue, Keith Kaye

## Abstract

**Background:** *Clostridioides difficile* infection (CDI) is a major source of morbidity and mortality. Even after recovery, recurrent CDI (rCDI) occurs frequently, and concomitant antibiotic use for treatment of a concurrent non–*C. difficile* infection is a major risk factor. Treatment with fidaxomicin versus vancomycin is associated with similar rate of cure and lower recurrence risk. However, the comparative efficacy of these 2 agents remains unclear in those receiving concomitant antibiotics. **Methods:** We conducted a randomized, controlled, open-label trial at the University of Michigan and St. Joseph Mercy hospitals in Ann Arbor, Michigan. Patients provided written informed consent at enrollment. We included all hospitalized patients aged ≥18 years with a positive test for toxigenic *C. difficile*, >3 unformed stools per 24 hours, and ≥1 qualifying concomitant antibiotic with a planned treatment of an infection for ≥5 days after enrollment. We excluded patients with complicated CDI, allergy to vancomycin–fidaxomicin, planned adjunctive CDI treatments, CDI treatment for >24 hours prior to enrollment, concomitant laxative use, current or planned colostomy or ileostomy, and/or planned long-term (>12 weeks) concomitant antibiotic use. Clinical cure was defined as resolution of diarrhea for 2 consecutive days maintained until the end of therapy and for 2 days afterward. rCDI was defined as recurrent diarrhea with positive testing within 30 days of initial treatment. Patients were randomized (stratified by ICU status) to fidaxomicin 200 mg twice daily or vancomycin 125 mg orally 4 times daily for 10 days. If concomitant antibiotic treatment continued >10 days, the study drug continued until the concomitant antibiotic ended. Bivariable statistics included *t* tests and χ^2^ tests. **Results:** After screening 5,101 patients for eligibility (May 2017–May 2021), 144 were included and randomized (Fig. 1). Study characteristics and outcomes are noted in Table 1. Baseline characteristics were similar between groups. Most patients were aged <65 years, were on a proton-pump inhibitor (PPI), and were not in the ICU. The mean duration of concomitant antibiotic was 18.4 days. In the intention-to-treat population, clinical cure (73% vs 62.9%; *P* =.195), and rCDI (3.3% vs 4.0%; *P* >.99) were similar for fidaxomicin and vancomycin, respectively. **Conclusions:** In this study of patients with CDI receiving a concomitant antibiotic, a numerically higher proportion were cured with fidaxomicin versus vancomycin, but this result did not reach statistical significance. Overall recurrence was lower than anticipated in both arms compared to previous studies in which duration of CDI treatment was not extended during concomitant antibiotic treatment. Future studies are needed to ascertain whether clinical cure is higher with fidaxomicin than vancomycin during concomitant antibiotic exposure, and whether extending the duration of CDI treatment reduces recurrence.

**Funding:** Merck & Co.

**Disclosures:** None

**Figure:**
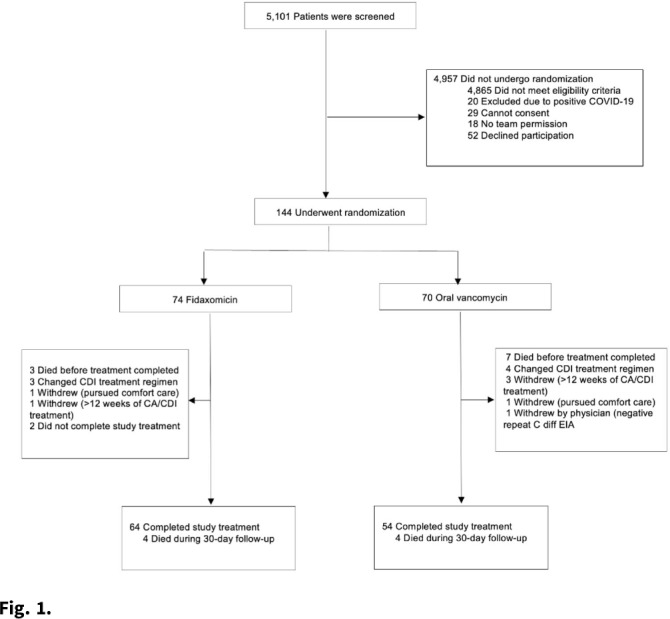

**Table:**